# Super-resolution reconstruction improves multishell diffusion: using radiomics to predict adult-type diffuse glioma IDH and grade

**DOI:** 10.3389/fonc.2024.1435204

**Published:** 2024-09-04

**Authors:** Chi Zhang, Peng Wang, Jinlong He, Qiong Wu, Shenghui Xie, Bo Li, Xiangcheng Hao, Shaoyu Wang, Huapeng Zhang, Zhiyue Hao, Weilin Gao, Yanhao Liu, Jiahui Guo, Mingxue Hu, Yang Gao

**Affiliations:** ^1^ Department of Radiology, Affiliated Hospital of Inner Mongolia Medical University, Hohhot, China; ^2^ MR Research Collaboration, Siemens Healthineers, Shanghai, China

**Keywords:** glioma, diffusion magnetic resonance imaging, deep learning, radiomics, diagnosis

## Abstract

**Objectives:**

Multishell diffusion scanning is limited by low spatial resolution. We sought to improve the resolution of multishell diffusion images through deep learning-based super-resolution reconstruction (SR) and subsequently develop and validate a prediction model for adult-type diffuse glioma, isocitrate dehydrogenase status and grade 2/3 tumors.

**Materials and methods:**

A simple diffusion model (DTI) and three advanced diffusion models (DKI, MAP, and NODDI) were constructed based on multishell diffusion scanning. Migration was performed with a generative adversarial network based on deep residual channel attention networks, after which images with 2x and 4x resolution improvements were generated. Radiomic features were used as inputs, and diagnostic models were subsequently constructed via multiple pipelines.

**Results:**

This prospective study included 90 instances (median age, 54.5 years; 39 men) diagnosed with adult-type diffuse glioma. Images with both 2x- and 4x-improved resolution were visually superior to the original images, and the 2x-improved images allowed better predictions than did the 4x-improved images (P<.001). A comparison of the areas under the curve among the multiple pipeline-constructed models revealed that the advanced diffusion models did not have greater diagnostic performance than the simple diffusion model (P>.05). The NODDI model constructed with 2x-improved images had the best performance in predicting isocitrate dehydrogenase status (AUC_validation=0.877; Brier score=0.132). The MAP model constructed with the original images performed best in classifying grade 2 and grade 3 tumors (AUC_validation=0.806; Brier score=0.168).

**Conclusion:**

SR improves the resolution of multishell diffusion images and has different advantages in achieving different goals and creating different target diffusion models.

## 1 Introduction

Following the identification and clarification of brain tumor pathogenesis and diagnostic and therapeutic processes, the World Health Organization released criteria for classifying tumors of the central nervous system in 2021 (1), which has further increased the clinical value of the convergence of tumor grade and molecular genotype. According to this classification system, most individuals diagnosed with adult-type diffuse glioma (approximately 80%) have poorer outcomes than patients with other types of glioma (2). The formulation of clinical decisions and prognosis prediction are dependent on the mutation status of isocitrate dehydrogenase (IDH), while an increase in tumor grade implies, to a certain extent that patients will require frequent radiotherapy and chemotherapy treatments, accompanied by a greater possibility of recurrence. MRI, the gold standard for preoperatively diagnosing glioma (3), may have potential as part of the development of an accurate method of predicting tumor pathology through imaging alone; such an approach could potentially optimize surgical decisions and improve clinical treatment strategies.

Previous studies have shown that, compared with conventional imaging, diffusion imaging can be used to capture additional brain tissue microstructural alterations and pathological changes caused by nuclear heterogeneity (4–6). Theoretically, advanced diffusion models, such as those based on neurite orientation dispersion and density imaging (NODDI) (7), itself based on the three-compartment model, and the mean apparent propagator (MAP) (8), which does not rely on *a priori* assumptions, should allow clinicians to better characterize the complexity and nonuniformity of the tissue microenvironment (9) and further improve the diffusion description of brain tissue over simple Gaussian diffusion models (such as diffusor tensor imaging [DTI]-based models). However, the findings of some clinical studies do not support these theoretical advantages (10, 11), reporting that these techniques may be limited by the spatial resolution of the acquired diffusion images (12). A high spatial resolution mitigates the partial volume effect, the phenomenon by which signal mixing occurs at the interfaces between different tissues. This enhancement facilitates more precise identification of boundaries between lesion areas and normal tissues, thereby improving diagnostic accuracy and reliability.

Super-resolution reconstruction (SR) is a technology through which the physical limitations of imaging systems can be overcome by generating high-resolution maps from one or more corresponding low-resolution images (13). SR methods are currently used in a variety of computer vision applications ranging from security and surveillance imaging (14) to object recognition (15). SR systems have also shown good applicability in the medical field. For example, they have been employed in the development of high angular resolution diffusion imaging brain templates from low angular resolution diffusion data from a single subject (16). Unlike Varentsova et al., Iglesias et al. (17) used SynthSR (a type of convolutional neural network) to synthesize higher spatial resolution images from portable low-field-strength MR images; notably, the high morphological correlation of different regions of interest in the brain demonstrated that SR was able to suitably improve the enhancement in the original image. In another study, a generative adversarial network (GAN)-based network architecture was used for quantitative analysis after migration (18), and the results suggested that the use of SR improved the diagnostic efficacy of radiomic models (which provide biological transformations of multiple feature matrices with more varied attempts for identifying imaging markers). Some scholars have also overcome the issue of inaccurate automatic glioma segmentation due to missing sequences or poor image quality through the combined application of U-Net and transfer learning (19). The use of SR is likely to increase the potential clinical applicability of multishell diffusion images through resolution improvement and facilitate the exploration of imaging markers for adult-type diffuse gliomas (**Supplementary Data Sheet 1**).

In this study, we attempted to use a GAN-based SR technique to improve the resolution of multishell diffusion images. We used a GAN for the following reasons: 1) Compared with other deep learning models, GANs perform adversarial training between the generator and discriminator to generate high-resolution images with richer details and more realistic textures than the original-resolution images. 2) The combination of content loss and adversarial loss enables GANs to consider both pixel-level accuracy and visual realism in performing super-resolution tasks. 3) The adversarial training mechanism of GANs easily adapts to highly complex image distributions and reduces the risk of overfitting. Two tasks (i.e., predicting the IDH status of adult-type diffuse glioma and predicting whether gliomas would be classified as grade 2 or 3) were subsequently performed with the constructed models to determine whether SR could be beneficial to clinical processes and to determine the practical applications of the diffusion models.

## 2 Materials and methods

We conducted a prospective study in accordance with the Declaration of Helsinki. The study was approved by our ethics committee (KY2023064), and all instances signed an informed consent form before enrollment.

We used the CLEAR checklist in the conduction of this study (20); it is included in this submission as **Supplementary Data**.

### 2.1 Instances and clinical data

In this study, we prospectively recruited consecutive instances who visited our institution between June 2018 and June 2023. Data from ninety instances (median age, 54.5 years; range, 21-77 years; 39 [43%] males) with a pathological diagnosis of adult-type diffuse glioma (in accordance with the 2021 WHO Classification of Tumors of the Central Nervous System) were used for image reconstruction and model building. Instances who were treated by methods including radiotherapy, chemotherapy, or concurrent radiotherapy and chemotherapy prior to pathology sampling and who had poor image quality were excluded. Previous studies (10) analyzed cohorts with overlapping data, in contrast to the present study, in which the scan-to-pathology time (median time of 4.5 days in this study) and the inclusion of new instances (n=13) were controlled and SR was employed more frequently.

### 2.2 Magnetic resonance scanning

All study instances underwent preoperative conventional MRI and diffusion imaging with a 3T scanner (MAGNETOM Skyra; Siemens Healthcare, Erlangen, Germany) equipped with a 32-channel head/neck coil.

The conventional MRI (cMRI) sequences included axial T1-weighted, axial T2-weighted, axial T2-weighted fluid-attenuated inversion-recovery (FLAIR), and 3D contrast-enhanced T1-weighted imaging, the last of which was performed after intravenous administration of 0.1 mmol/kg gadobutrol (Gadovist, Bayer AG, Berlin, Germany). The diffusion imaging sequences included axial diffusion-weighted imaging (DWI) and diffusion spectrum magnetic resonance imaging. The diffusion spectrum imaging scheme included the acquisition of a total of 128 diffusion samples, consisting of 16 b-values (200, 350, 400, 550, 750, 950, 1150, 1500, 1700, 1850, 1900, 2050, 2250, 2450, 2650 and 3000 s/mm2). The in-plane resolution was 2.65 mm. Detailed information on the parameters is provided in **Supplementary Table S1**.

### 2.3 Preprocessing

The diffusion parameters were calculated with Neuro-Diffusion Lab (NeuDiLab, Chengdu ZhongYing Medical Technology Co., Ltd., Chengdu, China), software developed in-house with Python based on the open-source tool DIPY (Diffusion Imaging in Python, https://dipy.org). The software is equipped with FSL-based brain extraction, eddy current and head motion correction, and smoothing functions and allowed acquisition of the final quantitative parametric maps for 25 features from the 4 diffusion models (listed below) and B0 maps. Three advanced models (diffusional kurtosis imaging (DKI), NODDI and MAP-MRI) and 1 simple model (DTI)) were constructed. The cMRI data were bias corrected with the N4ITK MRT bias correction module in 3D-Slicer. Coregistration of the cMRI data and diffusion parameter maps was performed with ANTs in 3D-Slicer. Default parameters were used for bias field correction and image registration.

### 2.4 Image SR and effect assessment

**Figure 1:**
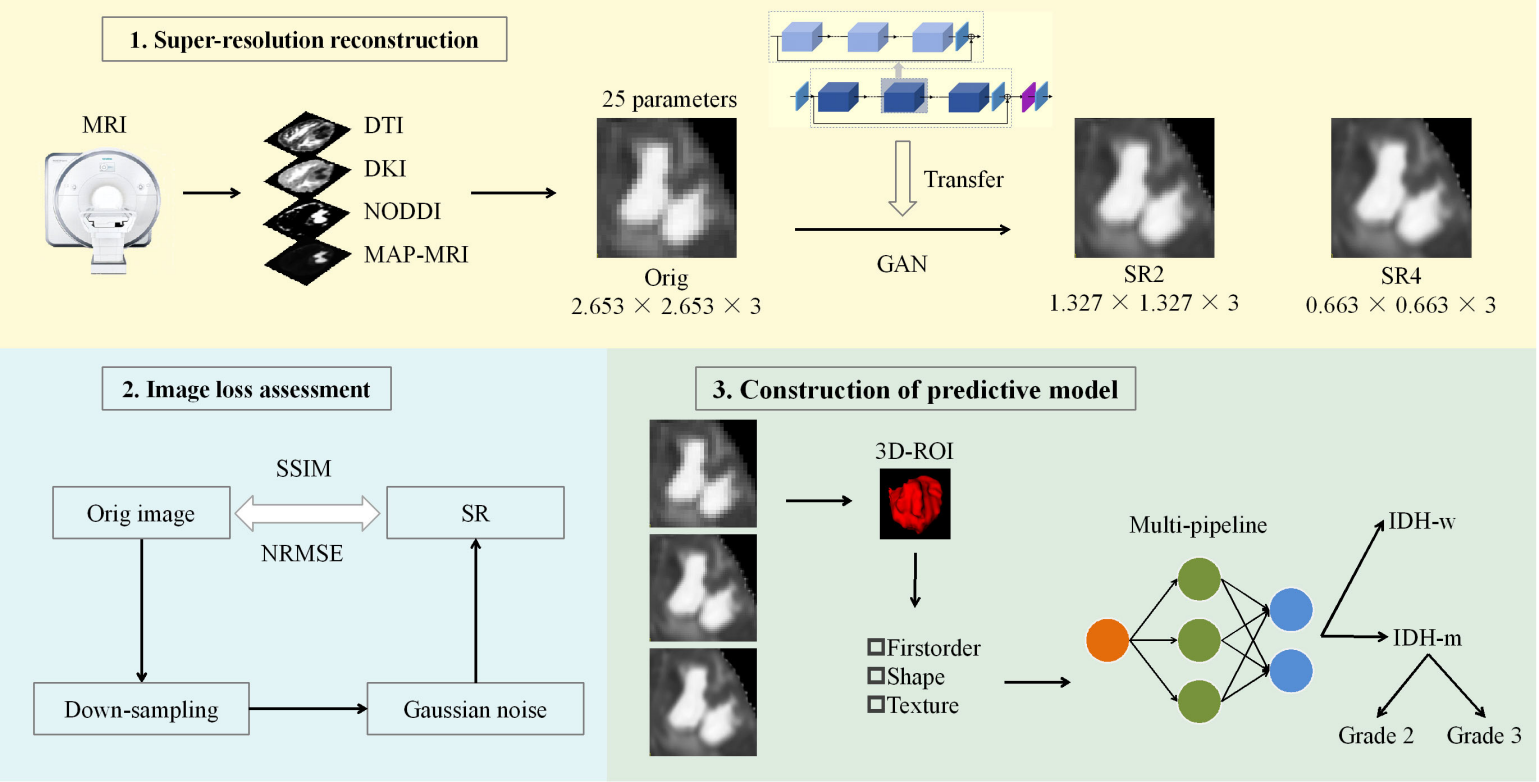
Workflow of the study. A GAN-based migration model was used for SR 25 parameter maps of the 4 diffusion models at two magnifications. After the reconstruction effect was evaluated, the actual clinical significance of the SR was judged via machine learning models constructed through multiple pipelines. ROC curve, decision curve and calibration curve analyses were used to evaluate and compare the performance of the models. DTI, diffusion tensor imaging; DKI, diffusion kurtosis imaging; NODDI, neurite orientation dispersion and density imaging; MAP, mean apparent propagation diffusion; GAN, generative adversarial network; SR2, super-resolution reconstruction 2x; SR4, super-resolution reconstruction 4x; SSIM, structural similarity; NRMSE, normalized root-mean-square error; ROI, region of interest; IDH-w, isocitrate dehydrogenase wild-type; IDH-m, isocitrate dehydrogenase mutant.

We performed SR on the original images with a pretrained GAN (https://github.com/OnekeyAI-Platform/onekey). The core of the migration model is a deep residual channel attention network (21) (https://github.com/XPixelGroup/BasicSR/blob/master/README_CN.md and https://github.com/yulunzhang/RCAN). Before training the GAN model, the input images underwent preprocessing to remove noise and artifacts, followed by normalization of the intensity values. The depth of the network is increased by the residual, which consists of several residual clusters with long jump connections. Each residual cluster contains several channel residual blocks with short jump connections. Each channel attention residual block consists of a simple residual block and a channel attention mechanism. Moreover, residual information allows rich low-frequency information to be bypassed via multiple jump connections so that the main network focuses on learning high-frequency information. The channel attention mechanism performs adaptive rescaling of channel features by accounting for the interdependence between channels to change the influence weights of different channel features on the reconstructed image. Finally, diffusion parameter maps with 2-fold and 4-fold spatial resolution enhancements are obtained. The flowchart of this study is shown in **Figure 1**.

The effect of SR was analyzed with visual characterization and image loss quantification. A physician (JL.H.) with 13 years of experience in neuroradiology and blinded to the clinical and pathological details of the instance but otherwise aware of the tumor diagnosis evaluated the SR-enhanced images by eye. Forced-choice pairwise comparisons were used to evaluate all samples, in which the sharpness, contrast, and both noise and artifacts were compared among the original, 2x resolution, and 4x resolution images. The images were quantitatively evaluated in three steps. First, we downsampled the original images 2x and 4x. Gaussian noise was subsequently introduced into the images. Finally, the image resolution was increased with a GAN-based transfer model, after which the loss between the original image and the newly generated images was calculated. We measured both structural similarity (SSIM) and the normalized root-mean-square error (NRMSE) for the whole brain and the tumor level (i.e., the solid tumor and peritumor edema regions).

### 2.5 Region of interest segmentation and feature extraction

Two physicians (P.W. and C.Z., with 4 and 3 years of neuroimaging experience, respectively) independently delineated regions of interest (ROIs) from the original images via a semiautomatic process in 3D-Slicer. Both radiologists were aware of the tumor diagnosis of the instance but were blinded to the clinical and pathological details. The ROIs were outlined on the B0 map while referring to the cMRI images. The solid tumor and peritumoral edema regions at all levels were selected as the ROIs; these regions are typically depicted radiologically as areas surrounded by T2-FLAIR abnormalities/high signals. For multicentric lesions, all regions were included in the ROIs. The ROIs for the 2x and 4x resolution images were obtained by upsampling the ROIs of the original images with the corresponding multiples, and then all the samples were examined to ensure that the extracted features corresponded to the same region as that in the original image. The Dice coefficient between the two physicians was 0.804; then the ROIs delineated by the more experienced physician was used for further feature extraction after review by another physician (Y.G.) with 28 years of experience in neuroradiology.

FeAture Explorer (FAE v0.5.8, https://github.com/salan668/FAE) (22) was used to extract radiomic parameters from the 3D-ROIs. Feature extraction was performed via PyRadiomics (version 3.0). In total, 107 features were extracted from the original images (**Supplementary Table S2**), including first-order features [n=18], shape-based features [n=14], gray level co-occurrence matrix features [n=24], gray level dependence matrix features [n=14], gray level run-length matrix features [n=16], gray level size-zone matrix features [n=16], and neighboring gray-tone difference matrix features [n=5]. The features were discretized by fixing the bin count (16 gray levels), and the remaining parameters were assigned the default configurations. Twenty-five sets of diffusion parameters were used for feature extraction; thus, 2675 features were extracted from the images at each resolution, for a total of 8025 features.

### 2.6 Model construction

Data were included in the training and internal test sets according to the instances’ enrollment time (23). Multiple pipeline combinations were then considered during model development, including 1 data balancing method (random upsampling), 3 feature normalization methods (mean, min–max and Z score normalization), 2 data dimensionality reduction methods (principal component analysis and Pearson correlation coefficients (cutoff = 0.85)), 4 feature selection methods (analysis of variance, recursive feature elimination, Kruskal-Wallis, and Relief), and 10 classifier methods (linear [logistic regression, logistic regression via least absolute shrinkage and selection operator, linear discriminant analysis, and support vector machine] and nonlinear [autoencoder, decision tree, random forest, AdaBoost, Gaussian process, and naïve Bayes]) (Scikit-Learn (version 0.24.2)) for a total of 240 basic pipelines. For pipelining, data balancing was used only for the training set, and feature scaling was independently applied to the validation and test sets. Ultimately, the number of features included in the model was restricted according to a rule of thumb; specifically, the maximum number of features was the number of instances in the training set divided by 10). The selection of the number of model features and hyperparameter tuning were performed via leave-one-out cross-validation. A time-independent internal test set that was not involved in the model selection was then used for model testing. Note that the division of the dataset was fixed, even for different pipelines.

For each imaging resolution for each task, 5 diagnostic models were selected from among the multiple pipelines, including 4 single-modal prediction models based on a single diffusion technique (DTI, DKI, MAP, and NODDI) and 1 fusion prediction model incorporating all the diffusion techniques; in this way, a total of 30 models (2 tasks × 3 image resolutions × 5 models) were selected for assessment.

### 2.7 Statistical analysis

Model performance was evaluated with receiver operating characteristic curve analysis. The output of the prediction models corresponds to the predicted probability of the task (ranging from 0 to 1). The results were then converted into binary predictions, where the threshold depends on the maximum Youden index. Model performance was compared in the cross-validation set through the integrated discrimination improvement metric; small sample sizes and an overreliance on the choice of p value and cutoff points limited the use of the DeLong test and net reclassification index (24). Calibration curves and Brier scores were used to assess deviations between the model results in the training set and the actual results. Decision curve analysis was used to assess the net clinical benefits of the different models under different threshold probabilities. The sample size calculations are presented in **Supplementary Data Sheet 1**.

Quantitative data are expressed as the means ± standard deviations. Student’s t test was used to compare instance ages, and the χ2 test, Fisher’s exact test or Mann-Whitney U test was used to compare categorical variables between groups. Comparisons between multiple groups were performed with one-way ANOVA, and the Bonferroni correction was used for p value adjustment for multiple comparisons. All the statistical analyses were two-sided, and P<.05 was used to indicate statistical significance. All the statistical analyses were performed with SPSS (version 24.0), R (version 4.3.1), and Python (version 3.9.18).

## 3 Results

### 3.1 Instance characteristics

In Task 1, seventy-two instances diagnosed between June 2018 and November 2021 were assigned to the training set; 28 had IDH-mutant glioma, and 44 had IDH-wild type glioma. Eighteen instances diagnosed between December 2021 and June 2023 were assigned to the internal test set, which included 6 with IDH-mutant glioma and 12 with IDH-wild type glioma (**Table 1**; **Supplementary Table S4**). Note that owing to sample size limitations, the dataset for Task 2 did not include the internal test set; rather, the data for all Grade 2 (n=12) and 3 (n=21) instances were used as the training set (**Supplementary Table S4**).

**Table 1:** Instance characteristics.

Variable	Full set
**Total number of instances**	90
2021 WHO Integrated Diagnosis (CNS WHO grade)	
Astrocytoma, IDH-mutant (2)	7
Astrocytoma, IDH-mutant (3)	7
Astrocytoma, IDH-mutant (4)	1
Oligodendroglioma, IDH-mutant and 1p/19q-codeleted (2)	5
Oligodendroglioma, IDH-mutant and 1p/19q-codeleted (3)	14
Glioblastoma, IDH-wild type (4)	56
Age (years)	
Median	54.5
Range	21-77
Sex	
Male	39
Female	51

Nine morphologic features (necrosis, cysticity, calcification, hemorrhage, tumor enhancement pattern, location, side, solid tumor border clarity, and edema) (10) were extracted from the imaging reports to be used as baseline features for within-set description and comparison. For the IDH predictions, only age (P=.001), necrosis (<.001), tumor location (<.001), and mode of enhancement (<.001) within the training set differed between the groups. For classifying grade 2 and grade 3 tumors, only tumor laterality (P=.032) within the training set differed between the groups. Because only a small number of baseline and morphological characteristics differed between the groups, no additional confounders were considered.

### 3.2 Image visualization and loss assessment

SR4 had the highest sharpness and contrast, followed by SR2 and the original image. SR4 also had the most amount of noise and highest number of artifacts; however, the patterns of noise and artifacts were not identical among the three sets of images.

**Figure 2:**
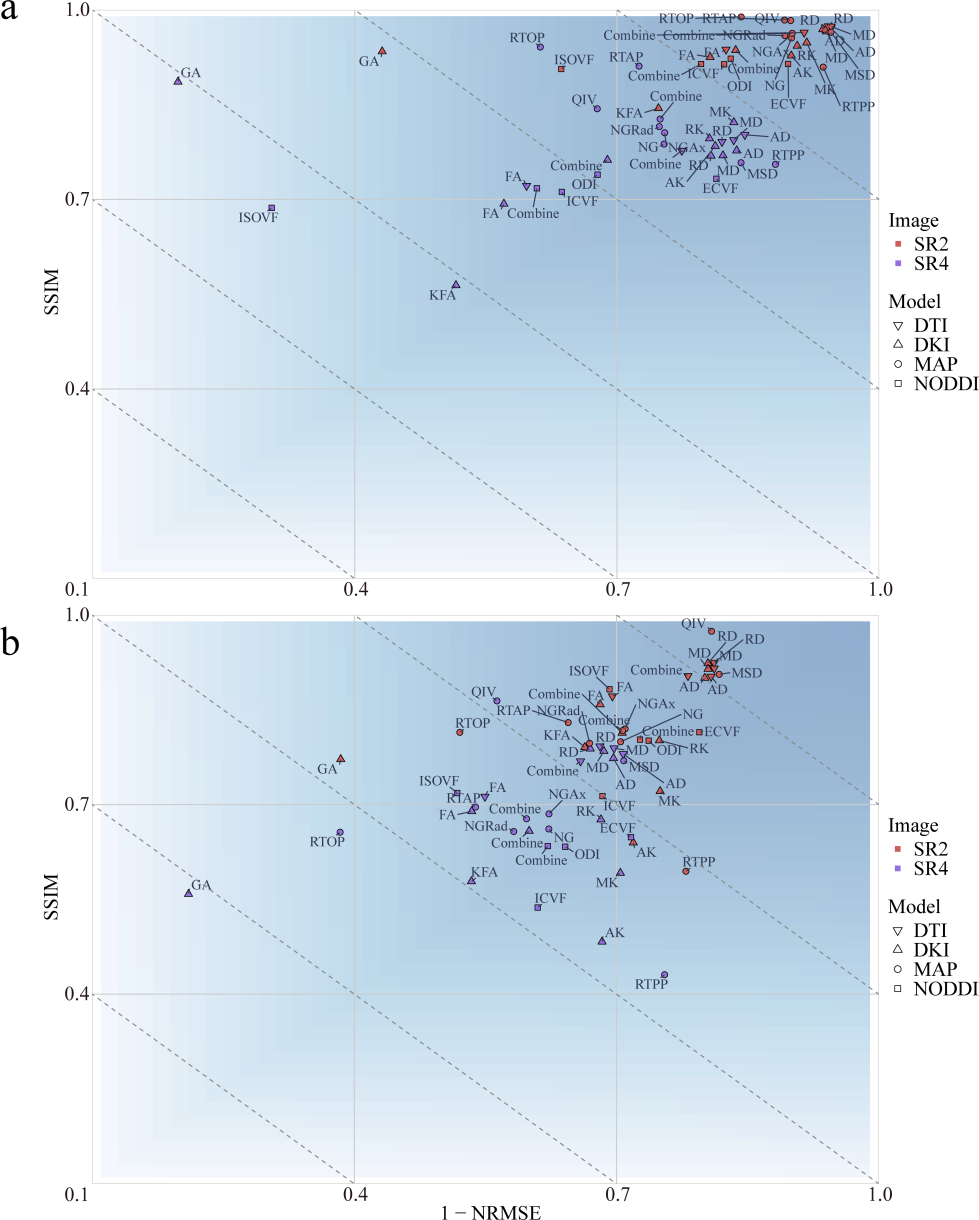
Mean loss for the multiple diffusion parameters corresponding to SR at different magnifications at the whole-brain **(A)** and tumor levels **(B)**. SSIM, structural similarity; NRMSE, normalized root-mean-square error; SR2, super-resolution reconstruction 2x; SR4, super-resolution reconstruction 4x; DTI, diffusion tensor imaging; DKI, diffusion kurtosis imaging; MAP, mean apparent propagation diffusion; NODDI, neurite orientation dispersion and density imaging.

The effects of image reconstruction were determined for both positive and negative SSIMs and NRMSEs. At both the whole-brain and the tumor region levels, the image loss in the 4 diffusion models constructed from the 2x-resolution images was less than that of the models constructed from the 4x-resolution images (P<.001) (**Figure 2**; **Supplementary Figure S1**). At the whole-brain level, SR2 corresponded to mean SSIM and NRMSE values of 0.827 and 0.278, respectively, whereas those of SR4 were 0.678 and 0.388, respectively. At the tumor region level, the mean SSIM and NRMSE for SR2 were 0.946 and 0.137, respectively, and those for SR4 were 0.778 and 0.291, respectively. In addition, the 4 diffusion models based on SR2 and the DKI and MAP models based on SR4 had greater image loss at the whole-brain level than at the tumor region (P<.001). The mean SSIM and NRMSE at the whole-brain level were 0.753 and 0.333, respectively, and those at the tumor region level were 0.862 and 0.214, respectively.

At the whole-brain level, the DTI achieved a smaller loss than did the other 3 diffusion models (P<.001). At the tumor region level, the loss with the NODDI was greater than that with the remaining 3 diffusion models (P<.001). At both the whole-brain and tumor-region levels, the fractional anisotropy (FA) loss was greater than that of the other 3 parameters in the DTI model (P<.001). No differences in parameter losses were observed for the other 3 diffusion models (P>.05).

### 3.3 Features correlated within different resolutions

According to Pearson correlation analysis, the correlation coefficient between the original images and the SR2 images ranged from -0.984 to 0.999 (**Supplementary Figure S2**), whereas that between the original image and SR4 ranged from -0.578 to 0.585. Radiomic features extracted from the original images were more highly correlated with the radiomic features extracted from the SR2 images than from those extracted from the SR4 images.

### 3.4 Comparisons of multiple pipelines and model selection

**Figure 3:**
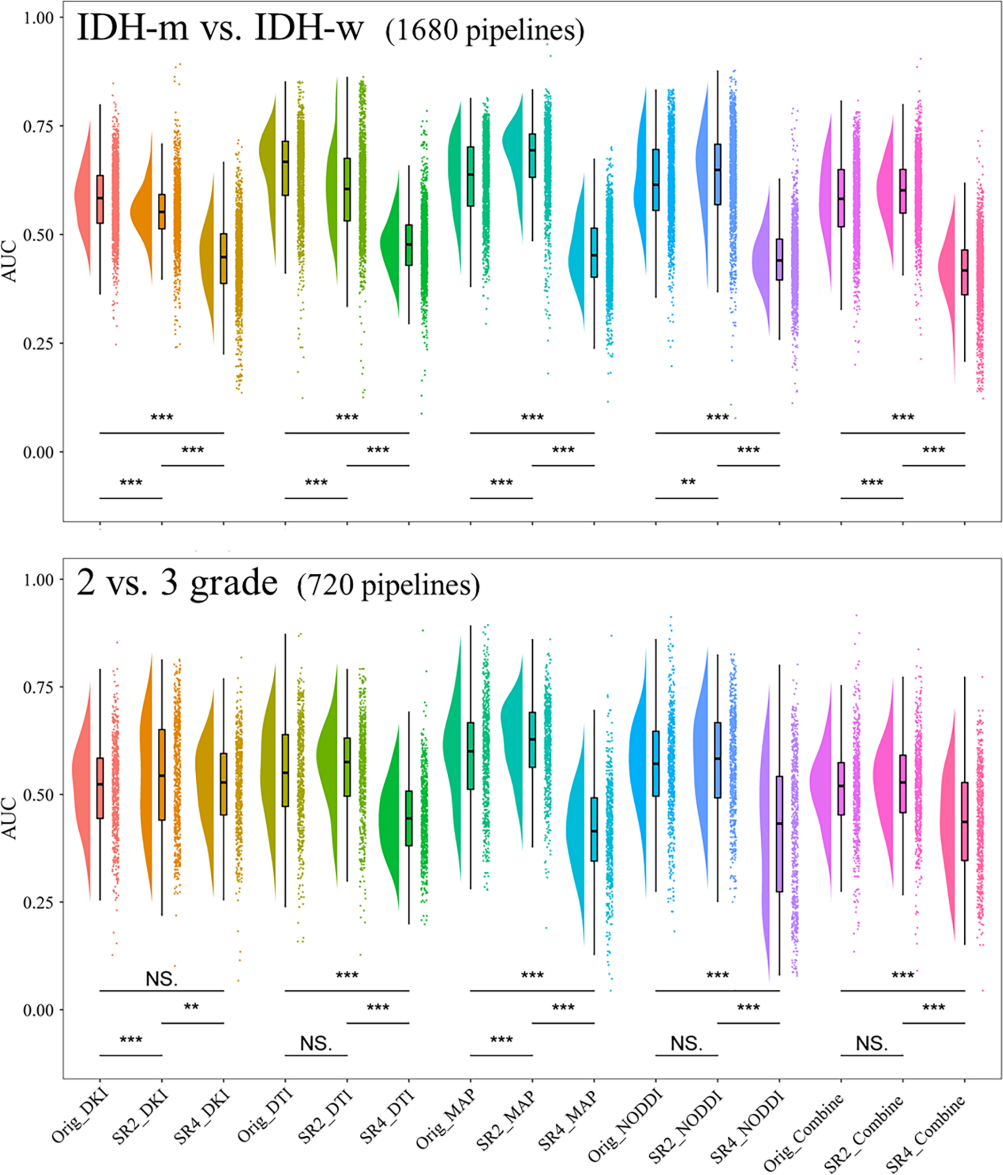
Comparison of the AUCs in the cross-validation sets of models constructed through different pipelines. **P <.01, ***P <.001. AUC, area under the curve; SR2, super-resolution reconstruction 2x; SR4, super-resolution reconstruction 4x; DTI, diffusion tensor imaging; DKI, diffusion kurtosis imaging; MAP, mean apparent propagation diffusion; NODDI, neurite orientation dispersion and density imaging. NS, No significance.

We considered a subgroup and a selected model to have high or low diagnostic efficacy when they exhibited the same trend within all the sets. The results showed that when predicting IDH, the SR4-based DTI prediction model had the lowest diagnostic performance across all sets (**Figure 3**; **Supplementary Figure S3**); when classifying Grade 2 and Grade 3 tumor classification, the performance did not seem to differ among the SR image sets.

**Figure 4:**
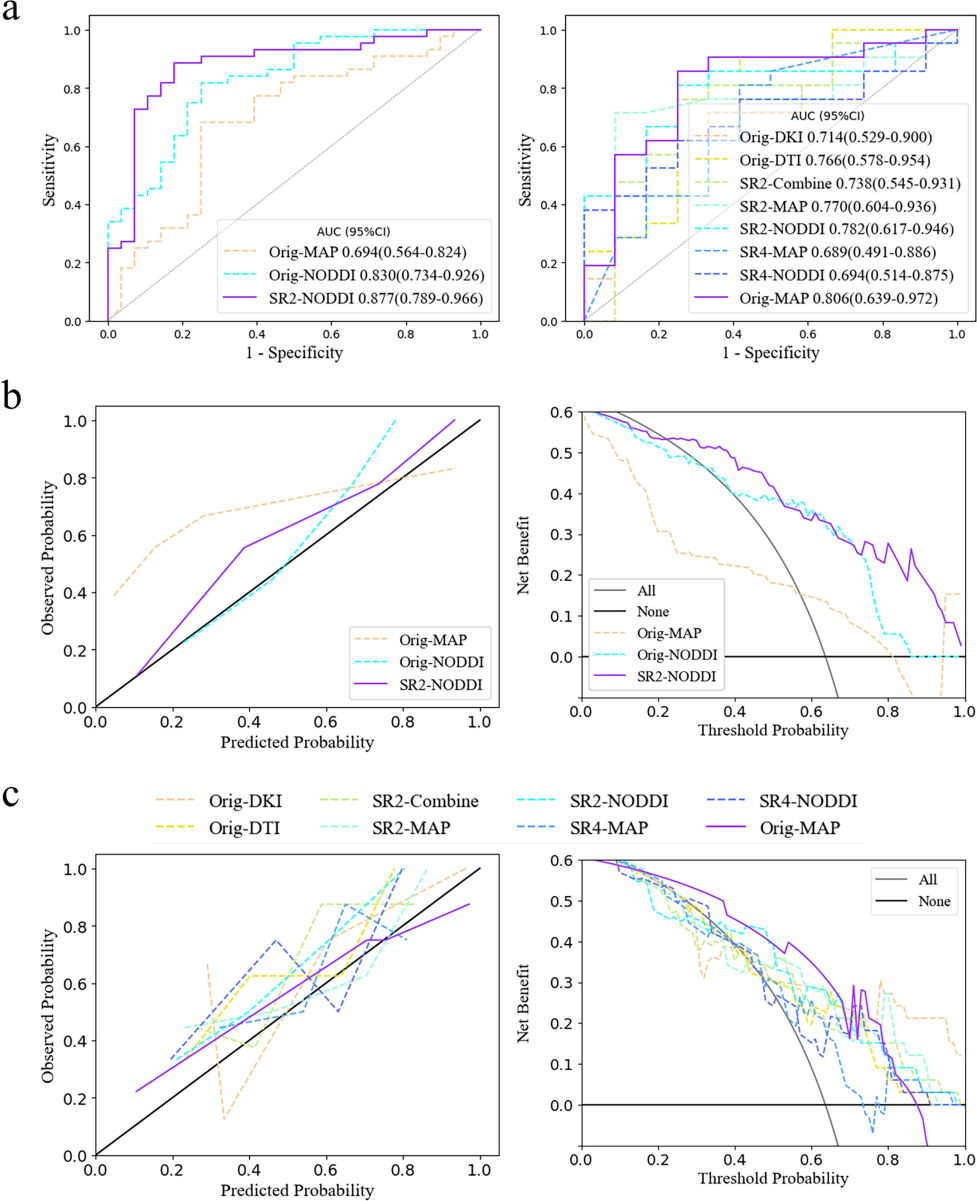
Model selection and clinical benefits. **(A)** ROC curves for the two tasks in the cross-validation set. The third and eight models had the highest diagnostic efficacy according to the integrated discrimination improvement index. **(B, C)** Calibration curve and decision curve analysis results in the training set in predicting IDH status and classifying tumors as grade 2/3. The models corresponding to the solid purple lines had the lowest Brier scores, 0.132 and 0.168, and their predictive ability was subsequently visualized via calibration curves (full Brier scores are provided in **Supplementary Table S5**). Additionally, these two models had the greatest net clinical benefits according to the decision curve analysis. AUC, area under the curve; SR2, super-resolution reconstruction 2x; SR4, super-resolution reconstruction 4x; DTI, diffusion tensor imaging; DKI, diffusion kurtosis imaging; MAP, mean apparent propagation diffusion; NODDI, neurite orientation dispersion and density imaging.

For each of the 15 predictive models, the areas under the ROC curve (AUCs) in the cross-validation set ranged from 0.593-0.877 and 0.607-0.861 separately for the two tasks. We then further selected the models with the highest diagnostic performance (P>.05) from the 30 models by comparing the integrated discrimination improvement metric in the cross-validation sets and identified three predictive IDH status models and eight models for classifying grade 2 and grade 3 tumors (**Table 2**; **Figure 4**). Models whose outputs were closer to the true results were then further selected according to the Brier scores (**Supplementary Table S5**). Finally, we found that the SR2-based NODDI model best predicted IDH status, while the original image-based MAP model performed best in classifying grade 2 and grade 3 tumors.

**Table 2:** Selected features for model construction.

Task	Feature origin (N)^a^	Pipeline^c^ (normalization/dimension reduction/feature selector/classifier)	Feature name
IDH-m vs. IDH-w	SR2-NODDI^b^ (N = 6)	Mean/PCA/KW/LR	PCA_feature_5
			PCA_feature_11
			PCA_feature_13
			PCA_feature_24
			PCA_feature_31
			PCA_feature_75
IDH-m vs. IDH-w	Orig-MAP^b^ (N = 6)	Minmax/PCC/Rel/NB	MSD_gldm_LargeDependenceLowGrayLevelEmphasis
			QIV_glrlm_LongRunHighGrayLevelEmphasis
			MSD_firstorder_Kurtosis
			QIV_gldm_SmallDependenceLowGrayLevelEmphasis
			RTOP_firstorder_Variance
			NG_glszm_LargeAreaLowGrayLevelEmphasis
IDH-m vs. IDH-w	Orig-NODDI^b^ (N = 4)	Z score/PCC/RFE/SVM	ICVF_firstorder_Energy
			ODI_firstorder_Skewness
			ODI_glcm_Correlation
			ODI_glszm_SmallAreaHighGrayLevelEmphasis
Grade 2 vs. grade 3	Orig-MAP^b^ (N = 2)	Minmax/PCC/Rel/NB	NG_gldm_LargeDependenceLowGrayLevelEmphasis
			QIV_firstorder_Kurtosis
Grade 2 vs. grade 3	Orig-DKI^b^ (N = 1)	Minmax/PCC/RFE/LR	MK_glszm_LargeAreaHighGrayLevelEmphasis
Grade 2 vs. grade 3	Orig-DTI^b^ (N = 2)	Minmax/PCA/ANOVA/LR	PCA_feature_1
			PCA_feature_40
Grade 2 vs. grade 3	SR2-Combine^b^ (N = 1)	Minmax/PCC/RFE/LDA	MK_glrlm_LongRunHighGrayLevelEmphasis
Grade 2 vs. grade 3	SR2-MAP^b^ (N = 2)	Mean/PCA/ANOVA/LR-Lasso	PCA_feature_2
			PCA_feature_41
Grade 2 vs. grade 3	SR2-NODDI^b^ (N = 2)	Zscore/PCA/KW/AE	PCA_feature_1
			PCA_feature_34
Grade 2 vs. grade 3	SR4-MAP^b^ (N = 2)	Minmax/PCA/KW/LDA	PCA_feature_6
			PCA_feature_36
Grade 2 vs. grade 3	SR4-NODDI^b^ (N = 2)	Zscore/PCA/RFE/LR	PCA_feature_18
			PCA_feature_36

a The total number of features in the group.

b We included 11 different modeling approaches for identifying IDH type and predicting grade 2 and 3 glioma.

c Pipeline for processing valid data features for modeling.

The NODDI model of SR2 was constructed via logistic regression and consisted of six features chosen after principal component analysis (**Supplementary Figure S4**), including principal components 13, 11, 5, 31, 24, and 75, the first and last of which had the highest and lowest mean absolute feature contribution values, respectively (**Supplementary Figure S5**). In addition, we determined the local SHapley Additive exPlanation (SHAP) values for individual samples. The AUCs of the model in the training and validation sets were 0.903 (0.832-0.975) and 0.877 (0.789-0.966), respectively (**Table 3**). In the internal test set, with pathological confirmation as the reference standard, 4 (66.7%) of 6 instances were correctly predicted as having the IDH-mutant type, and 6 (50%) of 12 instances were correctly predicted as having the IDH-wild type (**Supplementary Figure S6**). Overall, the model achieved a favorable AUC of 0.819 (0.576-1) in the internal test set. When the threshold probability was greater than 20%, the SR2-based NODDI provided greater net clinical benefits than did the other 2 models in predicting IDH status compared with the case where no predictive model was used (**Figure 4**).

**Table 3:** Optimal model performance for the two tasks.

Task	Training set	Cross-validation set	Internal test set
IDH-m vs. IDH-w			
AUC^*^	0.903 (0.832–0.975)	0.877 (0.789–0.966)	0.819 (0.576–1)
Sensitivity	0.932 (41/44)	0.886 (39/44)	0.5 (6/12)
Specificity	0.786 (22/28)	0.821 (23/28)	0.667 (4/6)
PPV	0.872 (41/47)	0.886 (39/44)	0.75 (6/8)
NPV	0.88 (22/25)	0.821 (23/28)	0.4 (4/10)
ACC	0.875 (63/72)	0.861 (62/72)	0.556 (40/72)
Grade 2 vs. grade 3			
AUC^*^	0.814 (0.642–0.985)	0.806 (0.639–0.972)	–
Sensitivity	0.81 (17/21)	0.857 (18/21)	–
Specificity	0.75 (9/12)	0.75 (9/12)	–
PPV	0.85 (17/20)	0.857 (18/21)	–
NPV	0.692 (9/13)	0.75 (9/12)	–
ACC	0.788 (26/33)	0.818 (27/33)	–

Data in parentheses are the numerator/denominator of participants included for each parameter, unless otherwise indicated. The values correspond to the optimal threshold according to the maximum Youden index.

^*^Data are the means (95% CI).

The original image-based MAP model was constructed with the naïve Bayes algorithm and consisted of 1 texture feature (non-Gaussianity), which had the highest mean absolute feature contribution value (0.35), and 1 first-order feature (q-space inverse variance). The AUCs of the model were 0.814 (0.642-0.985) and 0.806 (0.639-0.972) in the training and validation sets, respectively.

## 4 Discussion

In this study, we used an SR technique (GAN-based model migration) to improve the resolution of 25 quantitative parameter maps with 4 diffusion models at different magnifications and then constructed a multiparametric radiomic model to predict adult-type diffuse glioma IDH status and classify tumors into grades 2 and 3. Our study demonstrated that the diffusion MRI radiomic models noninvasively predicted IDH mutation status and tumor grade. The NODDI model based on SR2 images had the highest diagnostic efficacy (validation_AUC = 0.877) and stability (Brier value = 0.132) in predicting IDH mutation status; furthermore, the use of higher-resolution reconstructed images resulted in greater loss and a decrease in diagnostic performance. In addition, comparisons of the diffusion models indicated that advanced diffusion techniques were not always advantageous over DTI.

SR methods can be broadly divided into two categories: interpolation-based and learning-based techniques. Interpolation-based super-resolution reconstruction relies primarily on mathematical interpolation algorithms, such as nearest neighbor interpolation, bilinear interpolation, and bicubic interpolation. These algorithms estimate the values of newly added pixels by calculating the mathematical relationships among existing pixels. However, the primary limitation of this kind of approach is that it cannot introduce new high-frequency information, leading to a certain degree of image smoothing and, consequently, image blurriness, particularly around image edges and in regions with complex textures. Furthermore, the enhancement in spatial resolution is relatively limited, making it difficult to meet the demands of high-precision applications. In contrast, learning-based techniques employ deep learning models to learn the mapping between low-resolution and high-resolution images (19). Compared with interpolation-based methods, learning-based techniques offer superior reconstruction quality, stronger generalization capabilities, and the ability to support arbitrary magnification factors. The 3D super-resolution reconstruction technology presented in this study has achieved reliable results in enhancing the spatial resolution of CT images, which has improved clinical predictions for atherosclerotic plaques (25) and lung tumors (26).

Traditional morphologic visual evaluation, radiomics, and deep learning approaches for assessing tumor heterogeneity through imaging involve qualitative and quantitative analyses within limited regions of the image, regardless of the image source (e.g., radiological, pathological, and so on). Images with high quality actually make this task easier, whereas those with low quality can lead to ‘incorrect’ outcomes, especially when the lesion area is small and noise is present. Many medical image studies (16–18) have demonstrated the clinical benefits of deep learning-based SR both qualitatively and quantitatively. However, as the resolution increases, the number of image artifacts and the amount of noise also increases. At this point, effective estimation and integration of blur models may be more important than the utilization of image priors (27), i.e., models that use high- and low-resolution images. Images generated with default fixed blur models may be too unclear or contain oversharpened artifacts. In our study, higher magnification resulted in more artifacts and greater noise, which we believe is one of the main reasons for the decrease in diagnostic model performance. Various methods, such as mean filtering, have been developed for removing noise from different sources during (28) and after image generation. We did not apply additional denoising in our study because the tumor region image losses remained low, and excessive image smoothing could reduce the ability to visualize tumor heterogeneity in the images. Additionally, the residual channel attention network used in the present study is effective at both denoising and enhancing the resolution of medical images (29).

In terms of diffusion model theory, non-Gaussian diffusion models can better describe pathophysiological states in the brain than Gaussian models can. This means that the results derived from DKI, NODDI, and MAP data should be better than those derived from DTI data. However, the theoretical specificity of diffusion models leads to different practical strengths for each model. For example, NODDI better captures microstructural changes resulting from white matter diseases by quantifying changes in neurite direction (30), whereas non-Gaussianity provides a more detailed assessment of diffusion characteristics than fiber bundle imaging does (9).

In this study, we did not obtain sufficient evidence to support the hypothesis that advanced diffusion models offer greater clinical benefits than simple diffusion models. Studies by Gao et al. (11), Guo et al. (31), and Wang et al. (10) produced results similar to our results. However, some scholars (32–34) believe that the MAP and NODDI approaches can be used to better predict glioma heterogeneity or differentiate gliomas from metastatic tumors. Studies that drew the latter conclusion, however, used simple inferential statistics based on histogram averages of tumor entities or peritumoral edema without involving the extraction of additional features or the establishment of comprehensive models, and these limited attempts may not facilitate accurate conclusions. Notably, other factors, such as variations in the selection of regions of interest, may have contributed to the differences in the results (35). FA has been included in various prediction models in previous studies; however, in the present study, DTI exhibited high losses when FA achieved the best SR effect, which may be one of the reasons why the clinical benefits of DTI were not significantly improved.

The results of this study indicate that the combined application of super-resolution reconstruction and magnetic resonance diffusion imaging may provide a more comprehensive understanding of the pathological features of gliomas, including cell density, invasiveness, and vascular distribution, thereby providing strong support for an accurate diagnosis. Additionally, clearer preoperative visualization translates to more precise preoperative planning, especially with the current high reliance on intraoperative fiber tracking navigation. However, issues such as technical complexity and generalizability, data processing and storage, standardization and normalization, and patient safety and privacy protection limit the implementation of this technology in clinical practice. The establishment of proprietary deep learning models on the basis of specific data and theoretical improvements in diffusion models are necessary.

This study has several limitations. First, the prospective nature of the study limited the number of subjects that could be analyzed, and diagnostic model building was relatively limited due to possible overfitting issues with the integrated model. Instead, clinical features and imaging morphological features were used for assessing the effects of potential confounding factors. Further large-scale, multicenter studies are needed to validate the discriminative performance of these diffusion-based indicators, reducing potential prospective biases and issues related to insufficient statistical power. Second, the study lacked true high-resolution multishell diffusion images for evaluating the reconstruction effect of GAN-based super-resolution; instead, a downsampling method followed by the addition of noise was adopted. Although this is a commonly used analysis method, additional data augmentation or optimization of the model structure can improve the model’s generalization ability and reconstruction quality. Third, the number of diffusion models analyzed in the study was limited, and certain commonly used or higher-order diffusion models, such as the diffusion-weighted model and the continuous-time random walk model, were not included. Future studies should incorporate these common models when assessing the effects of SR. Fourth, the test-retest procedure for diffusion MRI was not attempted in the same instances, and the ROIs created by a small number of physicians may not be representative of all situations. However, in practice, retest procedures are limited in terms of time, cost, and instance tolerance.

In conclusion, we used GAN-based SR to improve the resolution of four diffusion models (DTI, DKI, NODDI, and MAP), allowing better visualization on multishell diffusion images and the possibility of quantitatively predicting IDH status and tumor grades 2 and 3 in adult-type diffuse glioma patients. Future work should include proprietary GAN model training and applications for specific diffusion models to further determine whether parameters fitted by multishell diffusion models can serve as imaging markers for adult-type diffuse glioma or other types of tumor.

## Data Availability

The datasets presented in this article are not readily available because data sharing that may reveal personally identifiable information about persons shall be done so carefully and in accordance with applicable laws and regulatory agencies. Requests to access the datasets should be directed to Yang Gao, 1390903990@qq.com.
