# Supplementary material for: Super-resolution reconstruction improves multishell diffusion: using radiomics to predict adult-type diffuse glioma IDH and grade
Source: Front Oncol. 2024 Sep 4;14:1435204. doi: 10.3389/fonc.2024.1435204 (PMC11408129; doi:10.3389/fonc.2024.1435204)
Supplement: Supplementary file 1 [file DataSheet1.docx]

Supplementary Material

# Supplementary Data

I. Evidence before this study

With no start time limit and an end date of July 1, 2024, we searched PubMed for publications that used spatial analysis based on medical imaging techniques to study the spatial heterogeneity of gliomas. We used the search terms (((artificial intelligence) OR (machine learning) OR (deep learning) OR (generative adversarial network) OR (super-resolution) OR (radiomics) OR (radiogenomic)) AND ((glioma) OR (glioblastoma)) AND ((diffusion magnetic resonance imaging) OR (diffusion kurtosis) OR (diffusion tensor) OR (mean apparent propagation) OR (neurite orientation dispersion and density))) without language restrictions. We identified 103 original studies that applied machine learning to predict histological grade or associated genetic features or that highlighted the potential and advantages of machine learning methods for the preoperative prediction of pathological features in gliomas; of these, one-third reported the value of using machine learning methods with MRI for glioma grading.

Despite encouraging preliminary results, the clinical applicability of these methods remained unclear owing to the retrospective nature of these studies, the lack of validation, and flawed analytical methods. In addition, the lack of publicly available codes for the algorithms undermined the transparency and reproducibility of these prediction systems. The retrieved typically focused on only one aspect of a histological or genetic trait. In 54% of the studies, only conventional MRI was employed for exploratory analysis, and other functional imaging or other imaging data were not included. However, the application of multimodal data facilitated the development of comprehensive diagnostic models, such as diffusion models or perfusion models. Only 6 original studies involved the simultaneous analysis of 2 diffusion models. To our knowledge, no study has prospectively validated the potential benefits of combining radiomic, clinical, and imaging features with multiple diffusion models to improve the performance of artificial intelligence models in predicting the grade of adult-type diffuse glioma.

II. Sample size and power calculations

Approximately 70 subjects were consecutively enrolled in the study. To test hypothesis 1 (H1), 70 subjects were needed for the study to achieve an AUC of 0.80 for the model at ~85% power and an alpha = 0.05 (two-sided). The sample size calculation was based on the following assumptions: 1) the AUC in the null hypothesis was 0.60, and 2) the positive cases accounted for 66% of the population. The sample size and power calculations were performed with PASS 2021 software.

# Supplementary Figures and Tables

## Supplementary Figures


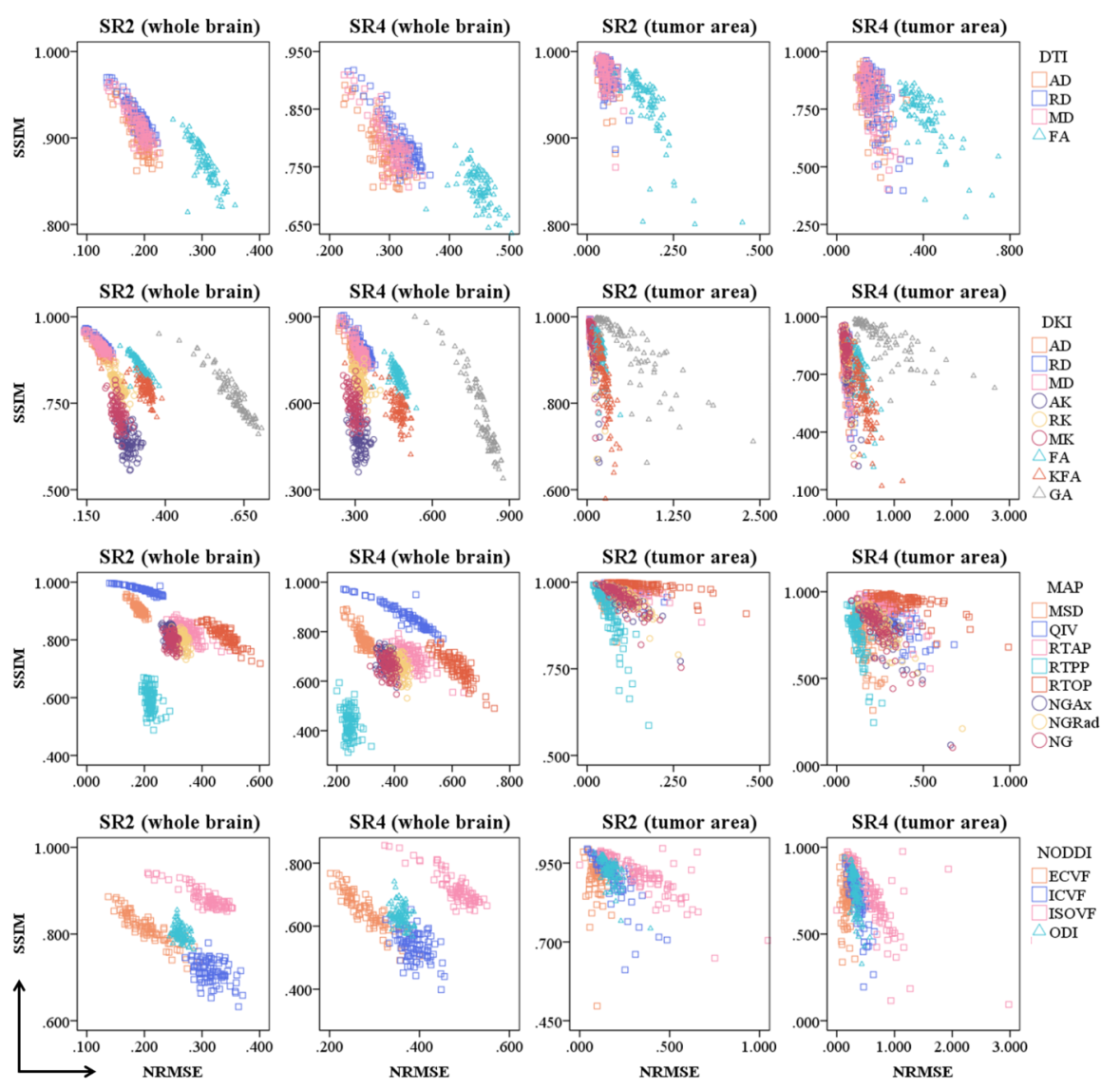


Figure S1. Image loss for multiple diffusion parameters corresponding to SR at different magnifications at the whole-brain and tumor levels for each instance

SSIM = structural similarity, NRMSE = normalized root-mean-square error, SR2 = super-resolution reconstruction 2x, SR4 = super-resolution reconstruction 4x, DTI = diffusion tensor imaging, DKI = diffusion kurtosis imaging, MAP = mean apparent propagation diffusion, NODDI = neurite orientation dispersion and density imaging, AD = axial diffusivity, RD = radial diffusivity, MD = mean diffusivity, AK = axial kurtosis, RK = radial kurtosis, MK = mean kurtosis, FA = fractional anisotropy, KFA = kurtosis fractional anisotropy, GA = generalized fractional anisotropy, MSD = mean squared displacement, QIV = q-space inverse variance, RTAP = return to the axis probability, RTPP = return to the plane probability, RTOP = return to the origin probability, NGAx = non-Gaussianity axial, NGRad = non-Gaussianity vertical, NG = non-Gaussianity, ECVF = extracellular volume fraction, ICVF = intracellular volume fraction, ISOVF = isotropic or free water volume fraction, ODI = orientation dispersion index.

**
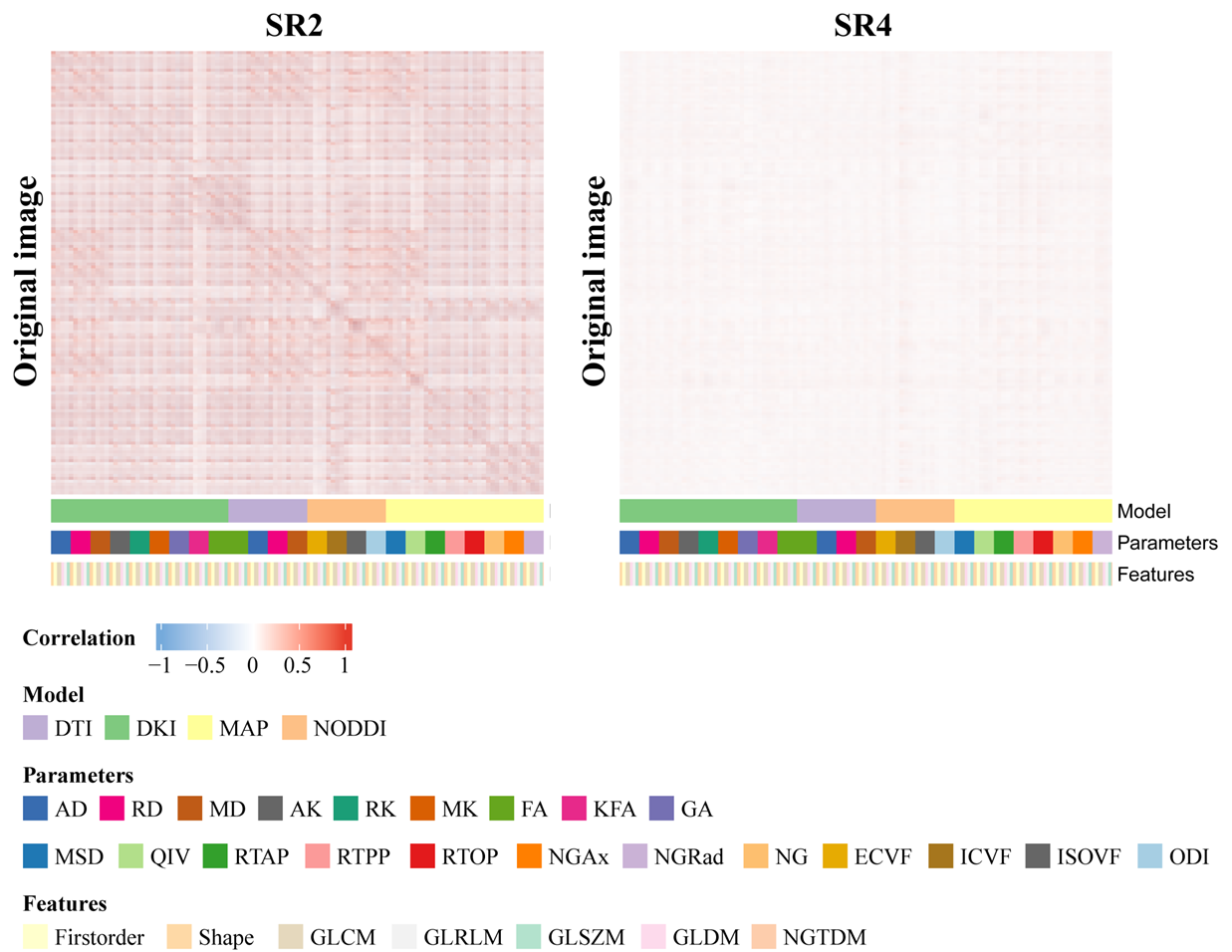
**

Figure S2. Pearson correlations between the original images and the super-resolution reconstructed images at different magnifications

SR2 = super-resolution reconstruction 2x, SR4 = super-resolution reconstruction 4x, DTI = diffusion tensor imaging, DKI = diffusion kurtosis imaging, MAP = mean apparent propagation diffusion, NODDI = neurite orientation dispersion and density imaging, AD = axial diffusivity, RD = radial diffusivity, MD = mean diffusivity, AK = axial kurtosis, RK = radial kurtosis, MK = mean kurtosis, FA = fractional anisotropy, KFA = kurtosis fractional anisotropy, GA = generalized fractional anisotropy, MSD = mean squared displacement, QIV = q-space inverse variance, RTAP = return to the axis probability, RTPP = return to the plane probability, RTOP = return to the origin probability, NGAx = non-Gaussianity axial, NGRad = non-Gaussianity vertical, NG = non-Gaussianity, ECVF = extracellular volume fraction, ICVF = intracellular volume fraction, ISOVF = isotropic or free water volume fraction, ODI = orientation dispersion index, GLCM = gray level cooccurrence matrix, GLRLM = gray level run length matrix, GLSZM = gray level size zone matrix, GLDM = gray level dependence matrix, NGTDM = neighborhood gray-tone difference matrix.


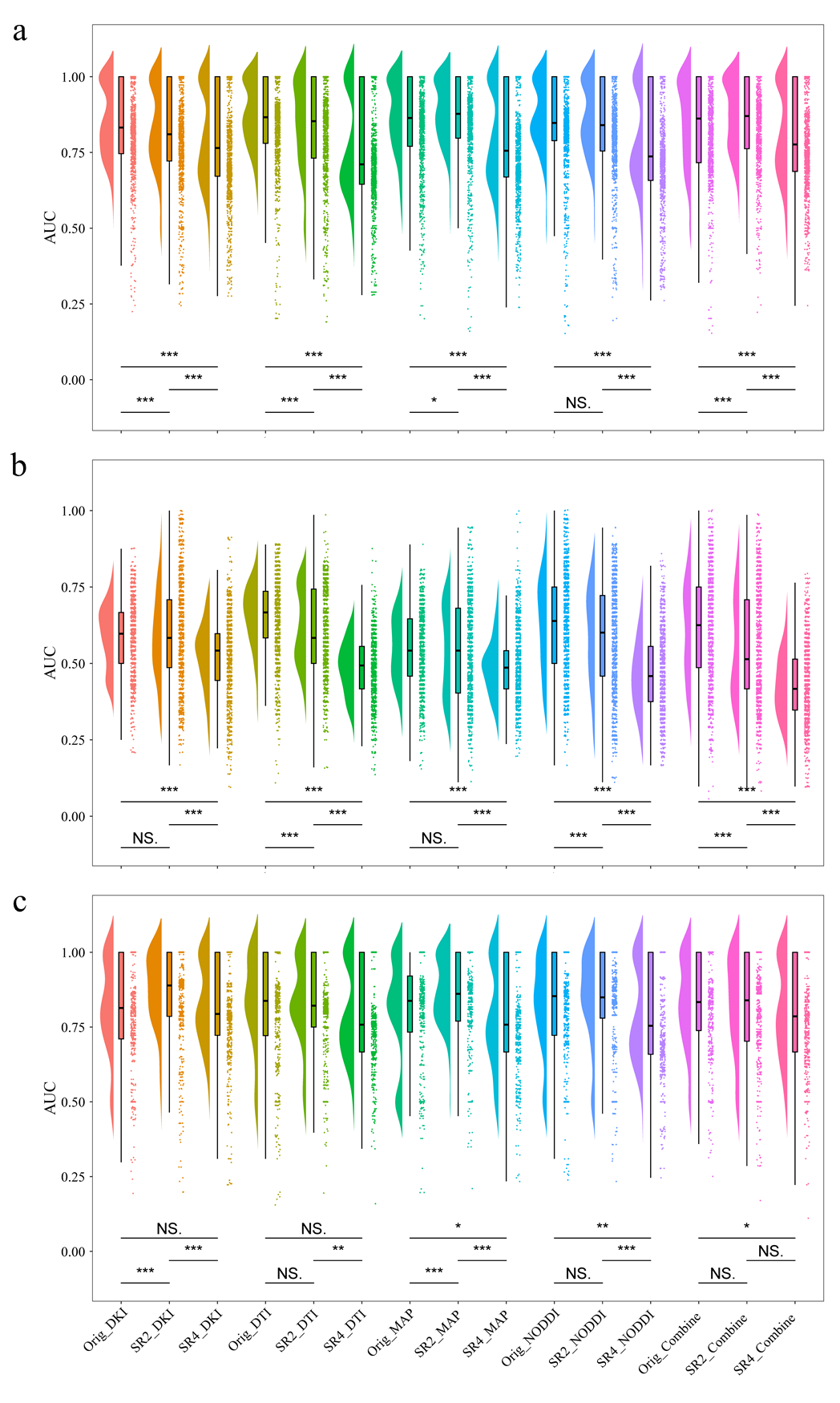


Figure S3. Visualization and comparison of the AUCs in the training set (a) and internal test set in predicting IDH status (b) and in the training set (c) for predicting tumor grade (grade 2 or 3)

*P < .05, **P < .01, ***P < .001.

AUC = area under the curve, SR2 = super-resolution reconstruction 2x, SR4 = super-resolution reconstruction 4x, DTI = diffusion tensor imaging, DKI = diffusion kurtosis imaging, MAP = mean apparent propagation diffusion, NODDI = neurite orientation dispersion and density imaging.


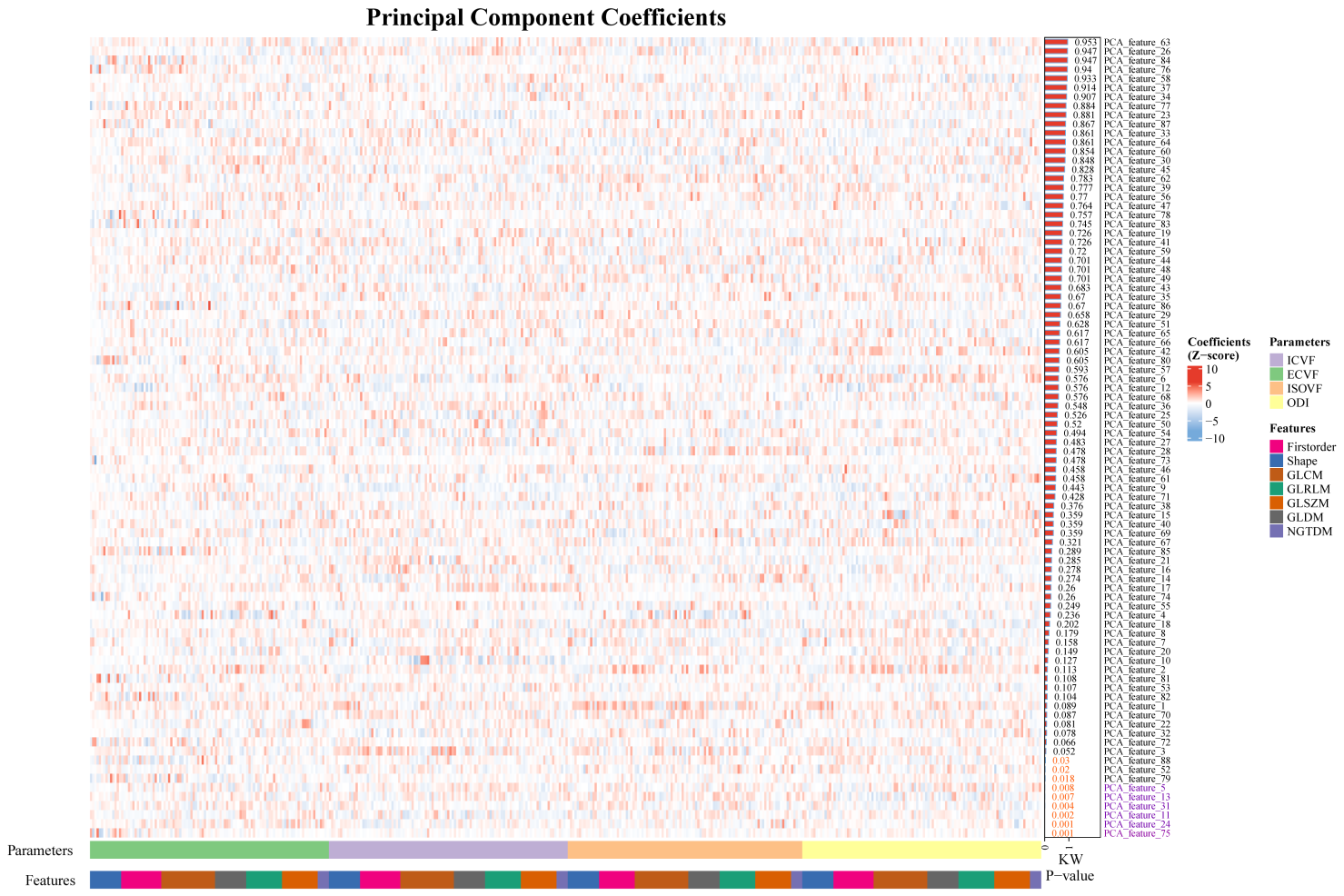


Figure S4. Dimensionality reduction and feature selection for the best model for predicting IDH status

Principal component coefficients among individual features and principal components were calculated via principal component analysis. Orange marks represent features with a p value less than .05 according to the Kruskal‒Wallis test. Purple marks represent the features included in the model.

ECVF = extracellular volume fraction, ICVF = intracellular volume fraction, ISOVF = isotropic or free water volume fraction, ODI = orientation dispersion index, GLCM = gray level cooccurrence matrix, GLRLM = gray level run length matrix, GLSZM = gray level size zone matrix, GLDM = gray level dependence matrix, NGTDM = neighborhood gray-tone difference matrix.

**
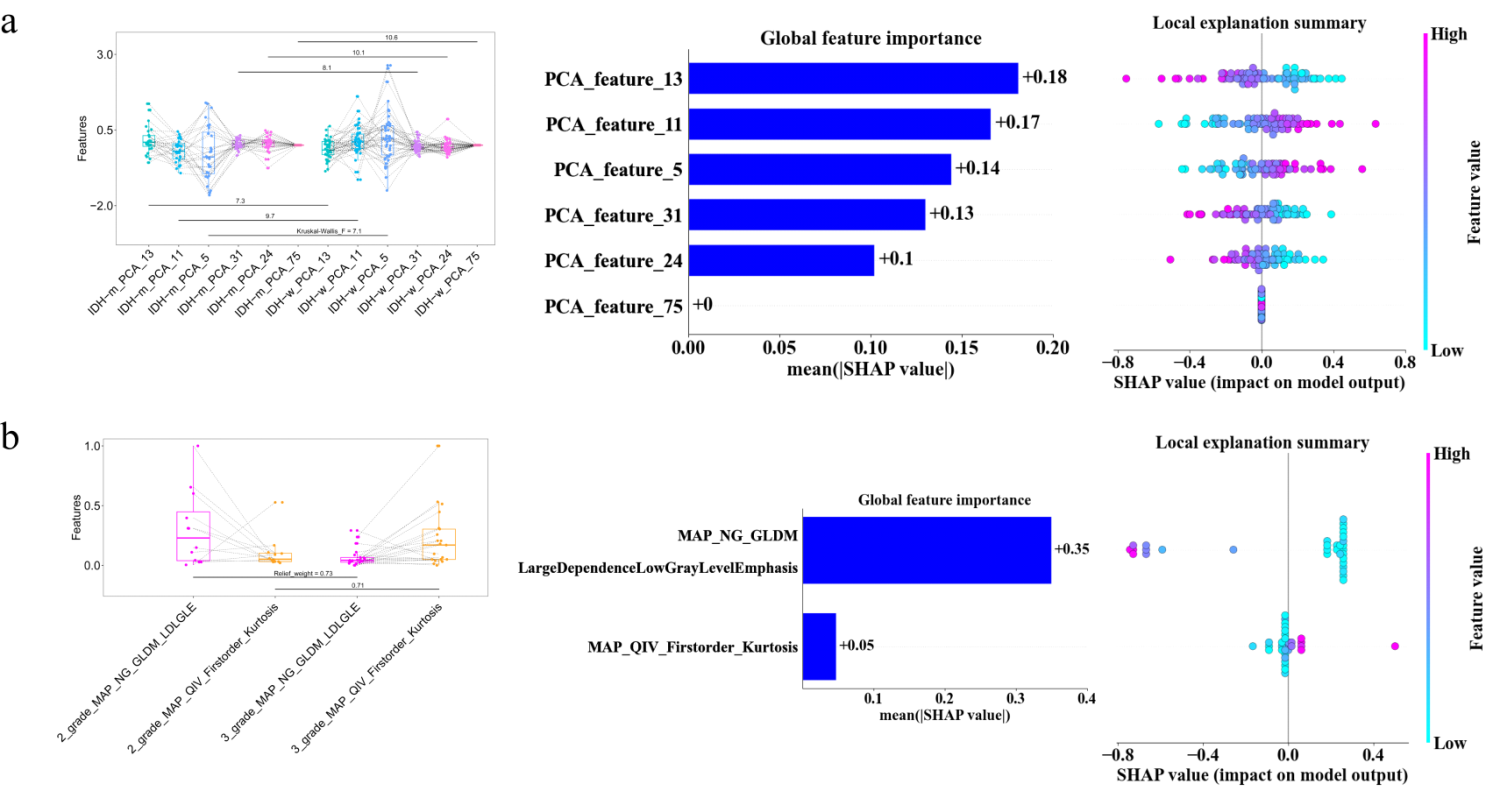
**

Figure S5. Distribution and contributions of the components used in the construction of the best models for predicting IDH status (a) and tumor grade (2 or 3) (b)

PCA = principal component analysis, MAP = mean apparent propagation diffusion, NG = non-Gaussianity, QIV = q-space inverse variance, GLDM = gray level dependence matrix.


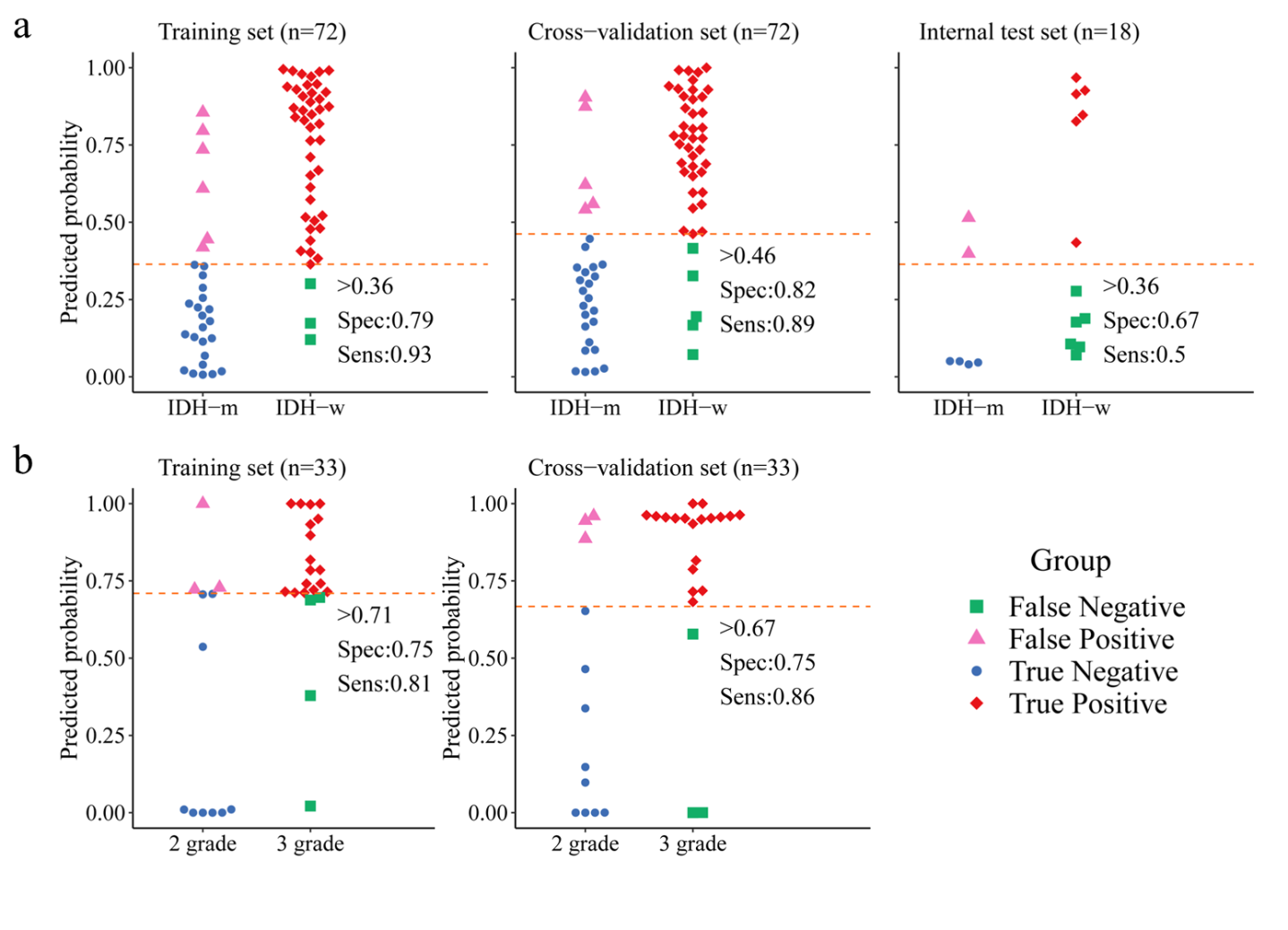


Figure S6. Profiles used to determine the best models for predicting IDH mutation status and classifying tumors as grade 2 or 3

All instances were identified as ‘IDH-m', ‘IDH-w', ‘grade 2’ or ‘grade 3' by the optimal model and assigned to four groups according to their true pathological responses in all sets. Using the optimal risk threshold, the sensitivity and specificity were determined.

IDH-m = isocitrate dehydrogenase mutant, IDH-w = isocitrate dehydrogenase wild-type, Spec = specificity, Sens = sensitivity.

## Supplementary Tables

Table S1. Anatomical and diffusion MRI parameters

|  | **T1** | **T2** | **T2-FLAIR** | **DWI** | **CE-T1** | **DSI** |
| --- | --- | --- | --- | --- | --- | --- |
| TA | 1 min 46 s | 1 min 45 s | 2 min 2 s | 1 min 4 s | 5 min 21 s | 15 min 40 s |
| TR (ms) | 1600 | 5500 | 6000 | 3530 | 2300 | 7000 |
| TE (ms) | 10 | 117 | 81 | 81、128 | 2.32 | 107 |
| FOV (mm^2^) | 230*230 | 230*230 | 230*230 | 230*230 | 240*240 | 220*220 |
| Base resolution | 320 | 320 | 320 | 160 | 256 | 98 |
| Phase resolution | 75 | 100 | 70 | 100 | 256 | 98 |
| Voxel size (mm^3^) | 0.4*0.4*5.5 | 0.7*0.7*5.5 | 0.7*0.7*5.5 | 1.4*1.4*5.5 | 0.9*0.9*0.9 | 2.2*2.2*3.0 |
| Diffusion mode | - | - | - | 3-Scan Trace | - | q-Space |
| b-value (s/mm^2^) | - | - | - | 0,1000 | - | 0-3000 |

CE-T1: Contrast-enhanced T1-weighted; these images were obtained last among all the scanning sequences.

DSI = diffusion spectrum MRI.

Table S2. Radiomic features extracted by FeAture Explorer

| **Feature Groups (N)^a^** | **Feature Names** | **Feature Groups (N)^a^** | **Feature Names** |
| --- | --- | --- | --- |
| First-order statistics^b^  (N = 18) | 10Percentile | GLCM^b^ (N = 24) | Autocorrelation |
|  | 90Percentile |  | ClusterProminence |
|  | Energy |  | ClusterShade |
|  | Entropy |  | ClusterTendency |
|  | InterquartileRange |  | Contrast |
|  | Kurtosis |  | Correlation |
|  | Maximum |  | DifferenceAverage |
|  | Mean |  | DifferenceEntropy |
|  | MeanAbsoluteDeviation |  | DifferenceVariance |
|  | Median |  | Id |
|  | Minimum |  | Idm |
|  | Range |  | Idmn |
|  | RobustMeanAbsoluteDeviation |  | Idn |
|  | RootMeanSquared |  | Imc1 |
|  | Skewness |  | Imc2 |
|  | TotalEnergy |  | InverseVariance |
|  | Uniformity |  | JointAverage |
|  | Variance |  | JointEnergy |
|  |  |  | JointEntropy |
|  |  |  | MCC |
|  |  |  | MaximumProbability |
|  |  |  | SumAverage |
|  |  |  | SumEntropy |
|  |  |  | SumSquares |
| Shape-based^b^ (N = 14) | Elongation | GLDM^b^ (N = 14) | DependenceEntropy |
|  | Flatness |  | DependenceNonUniformity |
|  | LeastAxisLength |  | DependenceNonUniformityNormalized |
|  | MajorAxisLength |  | DependenceVariance |
|  | Maximum2DDiameterColumn |  | GrayLevelNonUniformity |
|  | Maximum2DDiameterRow |  | GrayLevelVariance |
|  | Maximum2DDiameterSlice |  | HighGrayLevelEmphasis |
|  | Maximum3DDiameter |  | LargeDependenceEmphasis |
| Shape-based^b^ (N = 14) | MeshVolume | GLDM^b^ (N = 14) | LargeDependenceHighGrayLevelEmphasis |
|  | MinorAxisLength |  | LargeDependenceLowGrayLevelEmphasis |
|  | Sphericity |  | LowGrayLevelEmphasis |
|  | SurfaceArea |  | SmallDependenceEmphasis |
|  | SurfaceVolumeRatio |  | SmallDependenceHighGrayLevelEmphasis |
|  | VoxelVolume |  | SmallDependenceLowGrayLevelEmphasis |
| GLRLM^b^ (N = 16) | GrayLevelNonUniformity | GLSZM^b^ (N = 16) | GrayLevelNonUniformity |
|  | GrayLevelNonUniformityNormalized |  | GrayLevelNonUniformityNormalized |
|  | GrayLevelVariance |  | GrayLevelVariance |
|  | HighGrayLevelRunEmphasis |  | HighGrayLevelZoneEmphasis |
|  | LongRunEmphasis |  | LargeAreaEmphasis |
|  | LongRunHighGrayLevelEmphasis |  | LargeAreaHighGrayLevelEmphasis |
|  | LongRunLowGrayLevelEmphasis |  | LargeAreaLowGrayLevelEmphasis |
|  | LowGrayLevelRunEmphasis |  | LowGrayLevelZoneEmphasis |
|  | RunEntropy |  | SizeZoneNonUniformity |
|  | RunLengthNonUniformity |  | SizeZoneNonUniformityNormalized |
|  | RunLengthNonUniformityNormalized |  | SmallAreaEmphasis |
|  | RunPercentage |  | SmallAreaHighGrayLevelEmphasis |
|  | RunVariance |  | SmallAreaLowGrayLevelEmphasis |
|  | ShortRunEmphasis |  | ZoneEntropy |
|  | ShortRunHighGrayLevelEmphasis |  | ZonePercentage |
|  | ShortRunLowGrayLevelEmphasis |  | ZoneVariance |
| NGTDM^b^ (N = 5) | Busyness |  |  |
|  | Coarseness |  |  |
|  | Complexity |  |  |
|  | Contrast |  |  |
|  | Strength |  |  |

For the original images, 7 feature types were extracted, and a total of 107 features were obtained.

^a^: The total number of features in each group.

^b^: Includes morphological features, first-order histogram features and second-order features.

GLCM = gray level cooccurrence matrix, GLRLM = gray level run length matrix, GLSZM = gray level size zone matrix, GLDM = gray level dependence matrix, NGTDM = neighborhood gray tone difference matrix.

Table S3. Clinical and imaging morphometric characteristics of the instances in the sets used to predict IDH status

| **Variable** | **Training and cross-validation set (n=72)** | | | **Internal test set (n=18)** | | |
| --- | --- | --- | --- | --- | --- | --- |
|  | **IDH-m (n=28)** | **IDH-w (n=44)** | **P value** | **IDH-m (n=6)** | **IDH-w (n=12)** | **P value** |
| **Age (years)** | 47.54±12.04 | 57.05±10.77 | .001 | 49.00±8.92 | 55.58±10.77 | .102 |
| **Sex** |  |  | .999 |  |  | .620 |
| Male | 12/28 (42.86) | 18/44 (40.91) |  | 4/6 (66.67) | 5/12 (41.67) |  |
| Female | 16/28 (57.14) | 26/44 (59.09) |  | 2/6 (33.33) | 7/12 (58.33) |  |
| **Necrosis** |  |  | <.001 |  |  | .268 |
| Present | 14/28 (50) | 39/44 (88.64) |  | 3/6 (50) | 10/12 (83.33) |  |
| Absent | 14/28 (50) | 5/44 (11.36) |  | 3/6 (50) | 2/12 (16.67) |  |
| **Hemorrhage** |  |  | .086 |  |  | .999 |
| Present | 18/28 (64.29) | 37/44 (84.09) |  | 3/6 (50) | 7/12 (58.33) |  |
| Absent | 10/28 (35.71) | 7/44 (15.91) |  | 3/6 (50) | 5/12 (41.67) |  |
| **Calcification** |  |  | .137 |  |  | .529 |
| Present | 8/28 (28.57) | 6/44 (13.64) |  | 0/6 (0) | 2/12 (16.67) |  |
| Absent | 20/28 (71.43) | 38/44 (86.36) |  | 6/6 (100) | 10/12 (83.33) |  |
| **Cyst(s)** |  |  | .856 |  |  | .245 |
| Present | 26/28 (92.86) | 39/44 (88.64) |  | 6/6 (100) | 8/12 (66.67) |  |
| Absent | 2/28 (7.14) | 5/44 (11.36) |  | 0/6 (0) | 4/12 (33.33) |  |
| **Edema (≤1.5 cm)** |  |  | .055 |  |  | .999 |
| Yes | 20/28 (71.43) | 21/44 (47.73) |  | 6/6 (100) | 11/12 (91.67) |  |
| No | 8/28 (28.57) | 23/44 (52.27) |  | 0/6 (0) | 1/12 (8.33) |  |
| **Tumor borders** |  |  | .476 |  |  | .107 |
| Sharp | 16/28 (57.14) | 21/44 (47.73) |  | 2/6 (33.33) | 10/12 (83.33) |  |
| Blurry | 12/28 (42.86) | 23/44 (52.27) |  | 4/6 (66.67) | 2/12 (16.67) |  |
| **Tumor location** |  |  | <.001 |  |  | .316 |
| Frontal lobe or insula | 23/28 (82.14) | 14/44 (31.82) |  | 5/6 (83.33) | 6/12 (50) |  |
| Other | 5/28 (17.86) | 30/44 (68.18) |  | 1/6 (16.67) | 6/12 (50) |  |
| **Side** |  |  | .470 |  |  | .999 |
| Left | 13/28 (46.43) | 25/44 (56.82) |  | 3/6 (50) | 6/12 (50) |  |
| Right | 15/28 (53.57) | 19/44 (43.18) |  | 3/6 (50) | 6/12 (50) |  |
| **Enhancement** |  |  | <.001 |  |  | .084 |
| Patchy enhancement | 12/28 (42.86) | 4/44 (9.09) |  | 1/6 (16.67) | 1/12 (8.33) |  |
| Ring-like enhancement | 9/28 (32.14) | 39/44 (88.64) |  | 2/6 (33.33) | 10/12 (83.34) |  |
| No enhancement | 7/28 (25) | 1/44 (2.27) |  | 3/6 (50) | 1/12 (8.33) |  |

The data are presented as the mean ± standard deviation (SD) or n/N (%), where N is the total number of instances with available data. P values were calculated with the chi-square test, Fisher's exact test or the Mann‒Whitney U test. Underlined text indicates p values less than .05.

Table S4. Clinical and imaging morphometric characteristics of the instances in the sets used to predict tumor grade of 2 or 3

| **Variable** | **Training and cross-validation set (n=33)** | | |
| --- | --- | --- | --- |
|  | **Grade 2(n=12)** | **Grade 3 (n=21)** | **P value** |
| **Age (years)** | 43.17±9.94 | 50.14±11.88 | .063 |
| **Sex** |  |  | .300 |
| Male | 7/12 (58.33) | 8/21 (38.1) |  |
| Female | 5/12 (41.67) | 13/21 (61.9) |  |
| **Necrosis** |  |  | .071 |
| Present | 3/12 (25) | 13/21 (61.9) |  |
| Absent | 9/12 (75) | 8/21 (38.1) |  |
| **Hemorrhage** |  |  | .274 |
| Present | 6/12 (50) | 15/21 (71.43) |  |
| Absent | 6/12 (50) | 6/21 (28.57) |  |
| **Calcification** |  |  | .206 |
| Present | 1/12 (8.33) | 7/21 (33.33) |  |
| Absent | 11/12 (91.67) | 14/21 (66.67) |  |
| **Cyst(s)** |  |  | .999 |
| Present | 11/12 (91.67) | 20/21 (95.24) |  |
| Absent | 1/12 (8.33) | 1/21 (4.76) |  |
| **Edema (≤1.5 cm)** |  |  | .206 |
| Yes | 11/12 (91.67) | 14/21 (66.67) |  |
| No | 1/12 (8.33) | 7/21 (33.33) |  |
| **Tumor borders** |  |  | .999 |
| Sharp | 7/12 (58.33) | 11/21 (52.38) |  |
| Blurry | 5/12 (41.67) | 10/21 (47.62) |  |
| **Tumor location** |  |  | .643 |
| Frontal or insula | 9/12 (75) | 18/21 (85.71) |  |
| Other | 3/12 (25) | 3/21 (14.29) |  |
| **Side** |  |  | .032 |
| Left | 9/12 (75) | 7/21 (33.33) |  |
| Right | 3/12 (25) | 14/21 (66.67) |  |
| **Enhancement** |  |  | .133 |
| Patchy enhancement | 6/12 (50) | 7/21 (33.33) |  |
| Ringlike enhancement | 1/12 (8.33) | 9/21 (42.86) |  |
| No enhancement | 5/12 (41.67) | 5/21 (23.81) |  |

The data are presented as the mean ± standard deviation (SD) or n/N (%), where N is the total number of instances with available data. P values were calculated with the chi-square test, Fisher's exact test or the Mann‒Whitney U test. Underlined text indicates p values less than .05.

Table S5. Comparison of Brier scores in the training set

| **Task** | **Model** | **Brier score** |
| --- | --- | --- |
| IDH-m vs. IDH-w | SR2-NODDI | 0.132 |
| IDH-m vs. IDH-w | Orig-MAP | 0.320 |
| IDH-m vs. IDH-w | Orig-NODDI | 0.167 |
| Grade 2 vs. grade 3 | Orig-MAP | 0.168 |
| Grade 2 vs. grade 3 | Orig-DKI | 0.193 |
| Grade 2 vs. grade 3 | Orig-DTI | 0.204 |
| Grade 2 vs. grade 3 | SR2-Combine | 0.207 |
| Grade 2 vs. grade 3 | SR2-MAP | 0.195 |
| Grade 2 vs. grade 3 | SR2-NODDI | 0.197 |
| Grade 2 vs. grade 3 | SR4-MAP | 0.218 |
| Grade 2 vs. grade 3 | SR4-NODDI | 0.210 |

IDH-m = isocitrate dehydrogenase mutant, IDH-w = isocitrate dehydrogenase wild-type, NODDI = neurite orientation dispersion and density imaging, MAP = mean apparent propagation diffusion, DKI = diffusion kurtosis imaging, DTI = diffusion tensor imaging.
